# Fungal Spore-Feeding Thrips (Thysanoptera: Phlaeothripidae: Ldolothripinae) from Iran with Record of a Fourth Genus

**DOI:** 10.1673/031.011.5101

**Published:** 2011-04-18

**Authors:** Kambiz Minaei

**Affiliations:** Plant Protection Department, College of agriculture, Shiraz University, Shiraz, Iran.

**Keywords:** *Allothrips bournieri*, Fars province

## Abstract

The genus *Allothrips* Hood, with one species *A. bournieri* Mound, is reported for the first time in Iran and the generic classification of Phlaeothripidae is discussed briefly. A key is provided to distinguish the four genera recorded in Iran of the spore-feeding thrips in the ldolothripinae.

## Introduction

The family Phlaeothripidae, the only family in the suborder Tubulifera, comprises about 3000 described species (Mound 2010). A few genera are found typically in the flowers of Asteraceae and Graminae, and a few are predatory (Minaei and Mound 2008). Many genera, such as *Liothrips* and *Gynaikothrips*, include leaf feeding species some of which produce leaf or bud galls on a wide range of plants in the tropics (Mound and Marullo 1996). At least half of the species in this family are fungus feeders (mostly on hyphae) but with one major group, the Idolothripinae, feeding on spores (Mound and Palmer 1983; Morse and Hoddle 2006). Of the two subfamilies recognized in the family Phlaeothripidae (Idolothripinae and Phlaeothripinae), the spore feeding thrips are characterized by the presence of broad maxillary stylets and the absence in males both of glandular areas on sternite VIII and of short stout S2 setae on tergite IX. Broad maxillary stylets are considered to be a functional adaptation to feeding on fungal spores (Mound and Palmer 1983).

Among the 15 genera of Phlaeothripidae that have been recorded from Iran (Bhatti et al. 2009), three belong to the subfamily Idolothripinae (*Compsothrips, Megathrips, Pseudocryptothrips*), and the present paper is focused on this group. The remaining 11 genera belong to the subfamily Phlaeothripinae, in which the supra-generic classification is not satisfactory. Mound and Marullo (1996) recognize three major lineages within this subfamily, and in that arrangement four genera (*Dolicolepta, Haplothrips, Neoheegeria, Plicothrips*) belong to the *Haplothrips* lineage [= Tribe Haplothripini, in Mound and Minaei 2007); three genera (*Ataliothrips, Liothrips, Liophloeothrips*) belong to the *Liothrips* lineage; and five genera (*Cephalothrips*, Idiothrips, *Hoplandrothrips, Phlaeothrips, Stictothrips*) belong to the *Phlaeothrips* lineage. In spite of the many reports about the thysanopteran fauna of Iran since 1938 (Afshar 1938; Bhatti et al. 2009), and an increasing number of students in this country working on this group, only three species of Idolothripinae have so far been reported from Iran (Minaei and Alichi 2002). In this paper an additional genus is reported for the first time from Iran, and a key is provided to recognize the four genera of this group.

## Materials and Methods

The specimens of fungus-feeding thrips were collected from dead leaves of apple and oak using a Berlese funnel. When collected, the thrips specimens were removed using a fine brush and placed in vials. Microscopic slide mounts were prepared using a form of the protocol given in Mound and Kibby (1998). The photomicrographs were prepared using a Leica DM2500 Differential Interference Contrast microscope (www.leica-microsystems.com) and Automontage image processing software (www.syncroscopy.com/).

## Results

Although a large number of fungus feeding thrips, especially in the subfamily Idolothripinae, have been reported in other parts of the world (Mound and Marullo 1996; Mound and Palmer 1983; Priesner 1964a, 1964b; Reyes 1997), the fungivorous thrips fauna of Iran is not known well. The present investigation represents a small fraction of these thrips collected from some parts of Fars province.

***Allothrips bournieri*** Mound

*Allothrips pillichelus bournieri* Mound 1972: 35.

This species was described from southern France and Spain, but a series of both sexes has now been collected from leaf litter in Fars province, Iran. *Allothrips* species frequently produce quite large populations of apterae in leaf litter, but macropterae are rare. This probably leads to reduced gene flow between natural populations resulting in increased structural diversity between populations (Mound and Palmer 1983). As a result, a series of sub-species was proposed within the Old World species *A. pillichellus* (Priesner), and the New World species *A. megacephalus* Hood (Mound 1972). However, currently these subspecies are usually treated as full species (Mound 2010).

**Figure 1.**
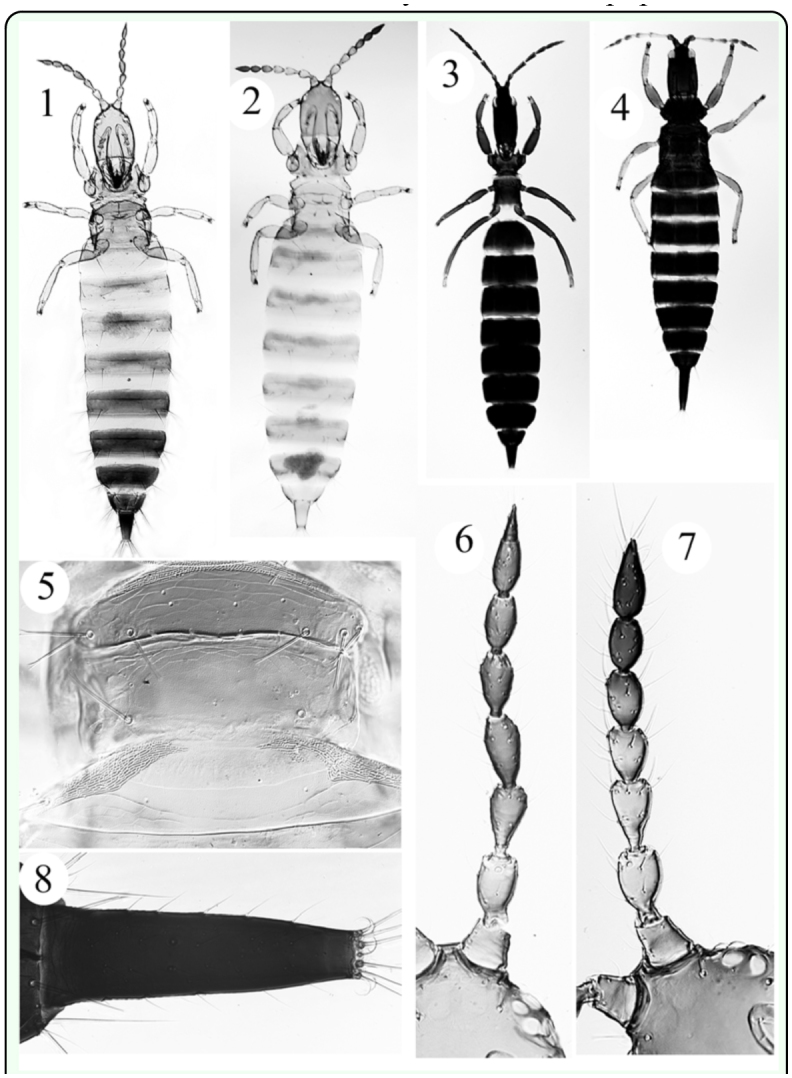
*Pseudocryptothrips meridionalis* ♀ **Figure 2.** *Allothrips bournieri* ♀ **Figure 3.** *Compsothrips reuteri* ♀ **Figure 4.** *Megathrips flavipes* ♀ **Figure 5.** *Allothrips bournieri* mesonotum **Figure 6.** *Pseudocryptothrips meridionalis* a part of head and right antenna **Figure 7.** *Allothrips bournieri* a part of head and right antenna **Figure 8.** *Megathrips flavipes* tube. High quality figures are available online.

**Diagnosis:** Female aptera; colour variable light brown and yellowish brown; antennal segments I–IV usually paler; tergites paler than head and pro thorax; major setae brown, sometimes pale at the apex; Head longer than wide; eyes reduced to four dorsolateral ommatidia; postocular setae long and capitate; mouth cone rounded maxillary stylets retracted to postocular setae, sub-parallel medially about one-third of head width apart; maxillary palps with large sensorium; major setae expanded; antennae 7-segmented (Figures 1, 7), segment III with 2 sensoria, VII narrower at base than VI at apex (Figure 7); foretarsal tooth absent; Pronotum with five pairs of major capitate setae well developed, epimeral sutures complete, prosternai basantra weak, ferna developed; mesonotum transverse and well developed with three pairs of expanded setae laterally (Figure 5) but two pairs of small finely pointed setae on posterior margin; mesopraesternum developed; metanotum without sculpture with one pair of long setae at the middle; pelta with a subbasal line of sculpture; tergites III–VIII with one pair of long marginal acute setae laterally; setae S1 and S2 on tergite IX expanded. Tube short, less than 1.5 times as long as basal width.

Male smaller and paler than female; a small foretarsal tooth present.

**Material examined:** Iran, Fars province, Badjgah, 6 ♀ from leaf litter of popular, 1l.viii.2008; 2 ♀, 1 ♂ from leaf litter of pistachio, 10.viii.2008; 3 ♀, 4 ♂ from leaf litter of popular (Moslem Behmanesh).

### Related and similar genera

*Allothrips* species live in leaf-litter on dead twigs in the tropics, subtropics and Mediteranean areas. The 700 species in almost 80 genera that comprise the subfamily Idolothripinae (Mound 2010) were assigned by Mound and Palmer (1983) to two Tribes, the Pygothripini and the Idolothripini. The first of these is divided into six subtribes including Allothripina. Two genera recorded from Iran (*Allothrips* and *Pseudocryptothrips*) belong to this subtribe that is distinguished by an apomorphy in the form of an enlarged terminal sensorium on the maxillary palps. *Allothrips* and *Pseudocryptothrips* are closely related, and apparently share the same habitat. In this study specimens of *P. meridionalis* usually were taken together with this *Allothrips* species.

**Key to genera and species of Idolothripinae from Iran**
1. Tube bearing long lateral setae (Figure 8); pelta with broad lateral lobes *Megathrips flavipes* (O. M. Reuter)
—. Tube without long lateral setae; pelta without broad lateral lobes 2
2. Body constricted at methothorax (cf. Figure 3); eyes with many facets dorsally; metanotum striate; head length more than twice its width *Compsothrips albosignatus* (O. M. Reuter)
— Body not constricted at methothorax (Figures 1, 2, 4); eyes with less than 10 facets dorsally (Figures 6, 7); metanotum smooth; head length at most 1.5 times its width 3
3. Antennae 8-segmented (Figure 6), segment IV with 4 sense cones *Pseudocryptothrips meridionalis* Priesner
— Antennae 7-segmented (Figure 7), segment IV with 2 sense cones *Allothrips bournieri* Mound

## Discussion

There are now 24 species recognized in *Allothrips* around the world (2 Africa, 5 South America, 4 USA, 2 Europe, 4 Australia, 3 India, 4 south eastern Asia) (Mound 2010). In Australia, the proportion of recorded Thysanoptera that are Idolothripinae is more than 18%, whereas in Iran it is about 2%. This partly is due to the study of thrips in Iran being restricted mainly to agricultural students, who work on groups with economic importance. The first thrips record in this country (Afshar 1938) confirms this statement. However, the Thysanoptera fauna of Iran is probably particularly poorly under-recorded. Most Iranian records of species in this order of insects have come from the Euro-Siberian (Northern) floristic region, or from the Irano Turanian (Central) floristic region. Almost no thrips are recorded from the southern Provinces along the southern coast, where the flora is different, and presumably the fauna is also likely to be richer.

## References

[bibr01] Afshar DJ (1938). *Pest of Summer Crops, Vegetables, Industrial Plants and Forages in Iran and Their Control*..

[bibr02] Bhatti JS, Alavi J, zur Strassen R, Telmadarraiy Z (2009). Thysanoptera in Iran 1938–2007: an overview.. *Thrips*.

[bibr03] Minaei K, Alichi M (2002). The first record of subfamily Idolothripinae (Thysanoptera: Phlaeothripidae) for Iran. Proceeding of the 15th Iranian Plant Protection Congress.

[bibr04] Minaei K, Mound LA (2008). The Thysanoptera Haplothripini (Phlaeothripidae) of Iran.. *Journal of Natural History*.

[bibr05] Mound LA (1972). Polytypic species of sporefeeding Thysanoptera in the genus *Allothrips* Hood (Phlaeothripidae).. *Journal of the Australian Entomological Society*.

[bibr06] Mound LA, Palmer JM (1983). The generic and tribal classification of spore-feeding Thysanoptera (Phlaeothripidae: Idolothripinae).. *Bulletin of the British Museum* (*Natural History) (Entomology*).

[bibr07] Mound LA, Marullo R (1996). *The Thrips of Central and South America: An Introduction*.. Memoirs on Entomology, International.

[bibr08] Mound LA, Kibby G (1998). Thysanoptera: An Identification Guide..

[bibr09] Morse JG, Hoddle MS (2006). Invasion biology of thrips.. *Annual Review of Entomology*.

[bibr10] Mound LA, Minaei K (2007). Australian insects of the Haplothrips lineage (Thysanoptera — Phlaeothripinae).. *Journal of Natural History*.

[bibr11] Mound LA (2010). Thysanoptera (Thrips) of the World — a checklist.. http://www.ento.csiro.au/thysanoptera/worldthrips.html.

[bibr12] Priesner H (1964a). A Monograph of the Thysanoptera of the Egyptian Deserts.. Publications de l'Institut du Desert d'Egypte.

[bibr13] Priesner H., Franz H. (1964b). Ordnung Thysanoptera (Fransenflugler, Thripse).. Bestimmungsbucher zur Bodenfauna Europas..

[bibr14] Reyes CP (1997). A catalogue of Philippine Thysanoptera (Insecta) (with Bibliography, 1776 to 1996).. *Emilio Aguinaldo College Research Bulletin*.

